# Supplementary material for: The AAA + ATPase TorsinA polymerizes into hollow helical tubes with 8.5 subunits per turn
Source: Nat Commun. 2019 Jul 22;10:3262. doi: 10.1038/s41467-019-11194-w (PMC6646356; doi:10.1038/s41467-019-11194-w)
Supplement: Supplementary file 1 — Supplementary Information [file 41467_2019_11194_MOESM1_ESM.pdf]

Supplementary Information

The AAA+ ATPase TorsinA polymerizes into hollow helical tubes with 8.5 subunits per turn

Demircioglu *et al.*

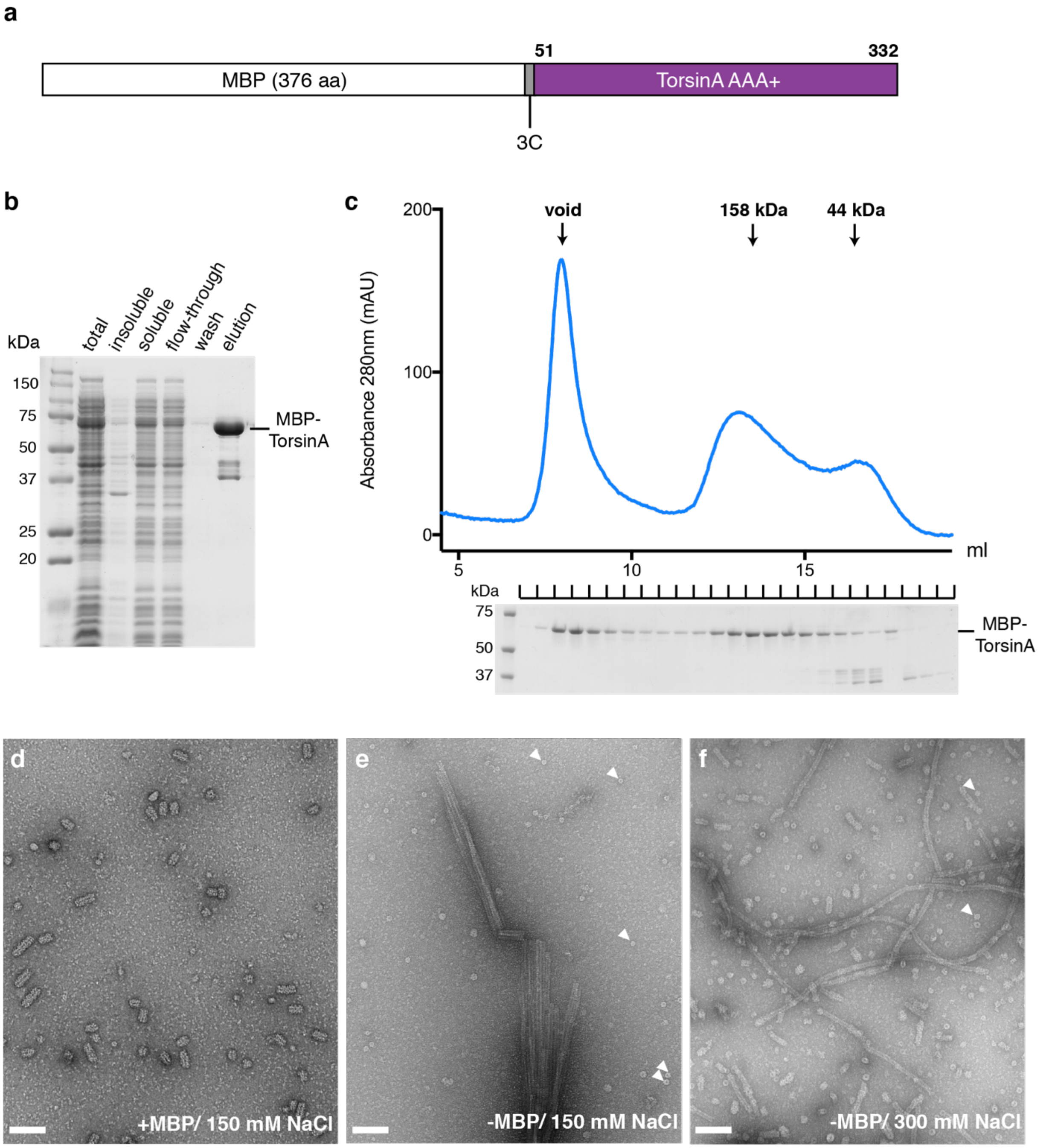

Supplementary Figure 1. Purification and biochemical characterization of TorsinA filaments

(**a**) Schematic diagram of the MBP-TorsinA construct used in the study. (**b**) SDS-PAGE analysis showing affinity purification steps of MBP-TorsinA on amylose resin. (**c**) Size exclusion

chromatography elution profile of MBP-TorsinA. The experiment was performed on a Superdex S200 HR10/300 column in running buffer containing 20 mM HEPES/NaOH pH 8.0, 150 mM NaCl, 10 mM $MgCl_2$, and 0.5 mM ATP. SDS-PAGE analysis of the fractions is shown below the chromatogram. (**d**) Negative stain electron micrograph of the MBP-TorsinA in running buffer described in (c). Short TorsinA filaments were observed regardless of whether the elute from the amylose resin or a void fraction from the gel filtration run was used. (**e**) Negative stain image of the TorsinA filaments after the MBP fusion tag was cleaved during dialysis against the same running buffer. The filaments grew considerably longer in size, however, they became clustered. (**f**) Same as (e), but with dialysis against a buffer identical to running buffer except containing 300 mM NaCl instead of 150 mM NaCl. Long and separated filaments, suitable for helical reconstruction, were obtained. Scale bar, 100 nm. Arrowheads mark the GroEL contamination observed in our amylose-affinity purified sample.

a

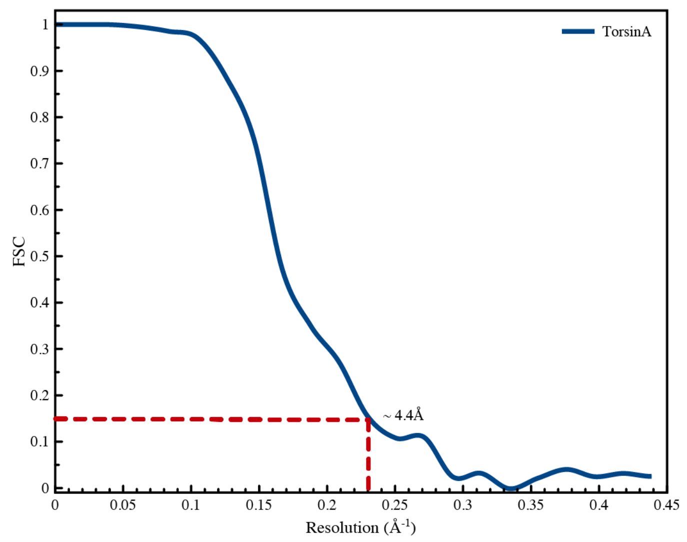

b

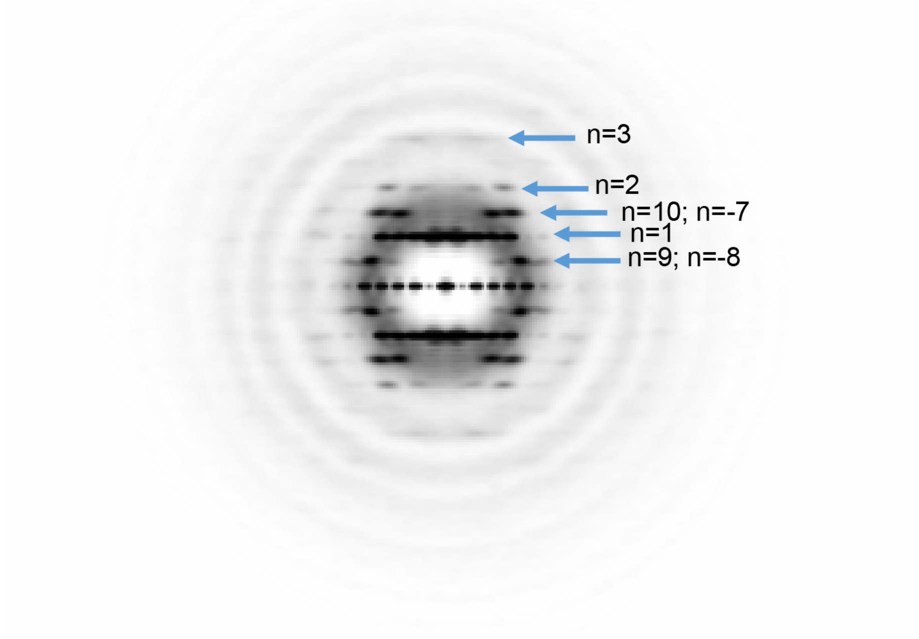

Supplementary Figure 2. Resolution estimate and power spectrum of TorsinA filaments

(**a**) The FSC between two-independent half maps, each generated from two non-overlapping datasets, shows a resolution of 4.4 Å at FSC=0.143. (**b**) The power spectra of 69,670 overlapping segments, which was used to determine the helical symmetry of the TorsinA filament.

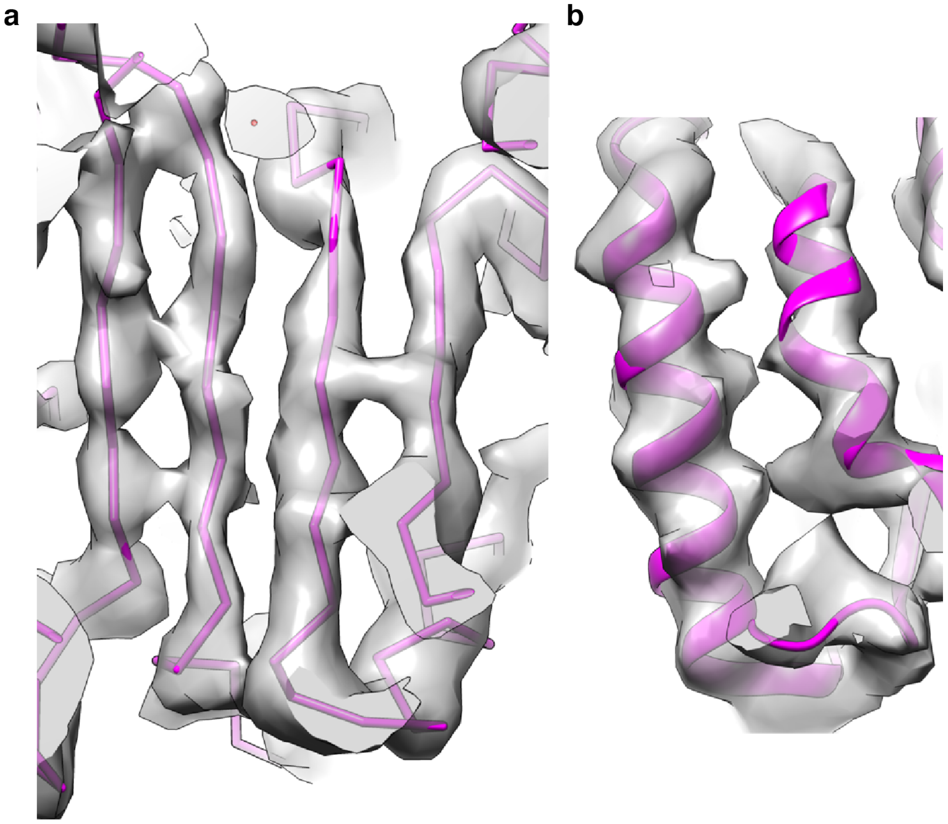

Supplementary Figure 3. Representative sections of the cryo-EM map

β-strands (**a**) and α-helices (**b**) of TorsinA are resolved in the map and we were able to build them unambiguously into the model at the resolution we obtained.

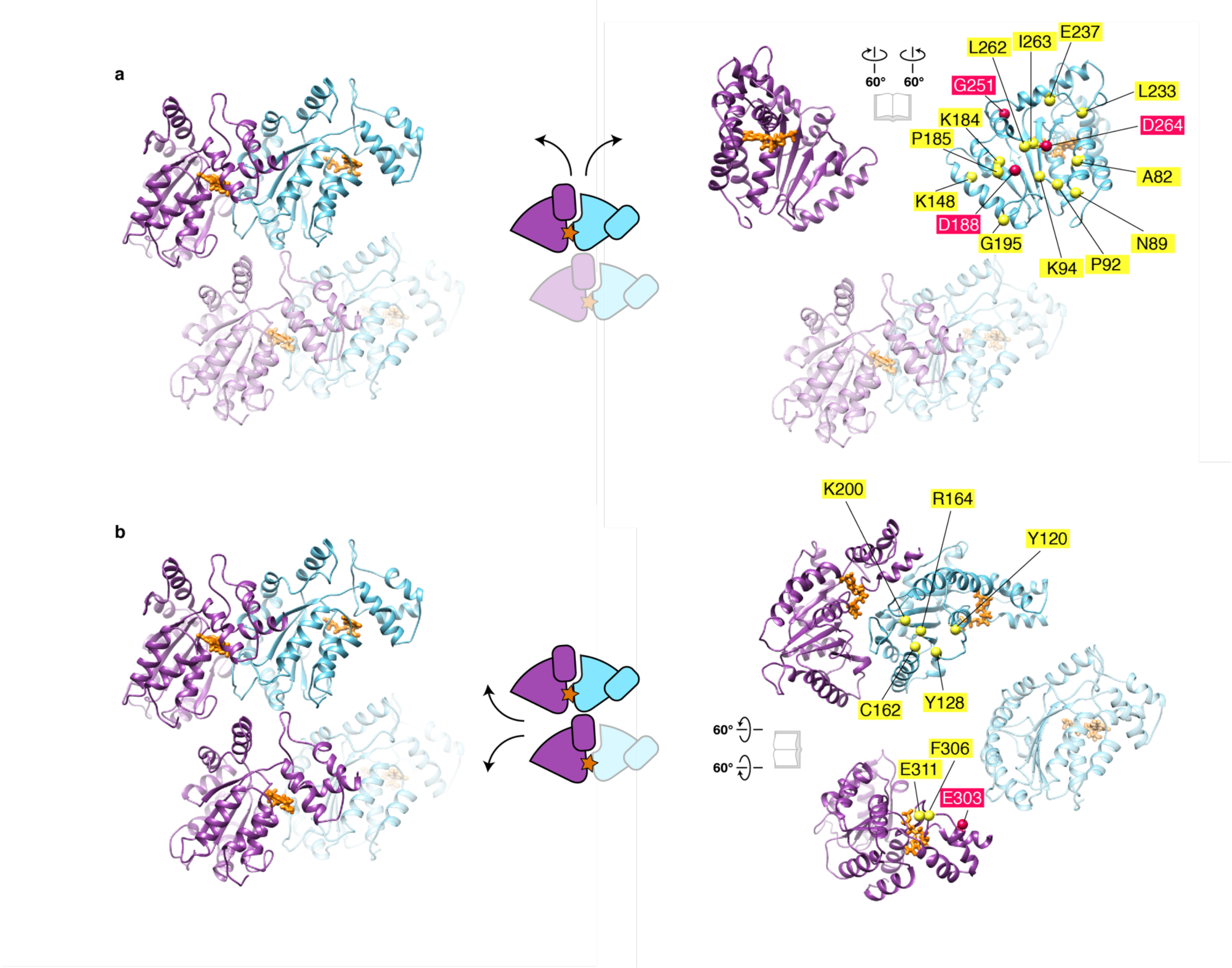
a
60°
60°
L262
I263
E237
G251
L233
K184
D264
P185
A82
K148
D188
G195
N89
K94
P92
b
K200
R164
Y120
C162
Y128
60°
60°
F306
E311
E303

Supplementary Figure 4. Analysis of the TorsinA-TorsinA interfaces within the filaments (**a**) A TorsinA-TorsinA dimer within a single helical turn is highlighted in full color (left), and schematically drawn (middle). An open-book representation of the interface between the two TorsinA protomers is shown (right). Conserved residues at the interface, on the non-catalytic face of TorsinA, are marked and labeled. The residues which have been mutated in this study to disrupt filament formation (see Supplementary Fig. 5) are shown as magenta colored dots. (**b**) Three TorsinA protomers interacting with each other between two adjacent helical turns are highlighted in full color (left), and schematically drawn (middle). The interface between the three molecules is shown in an open-book representation (right). All conserved residues which lie at the interface are marked and labeled. Note that the dystonia-relevant residue E303, colored in magenta, is one of the interface residues.

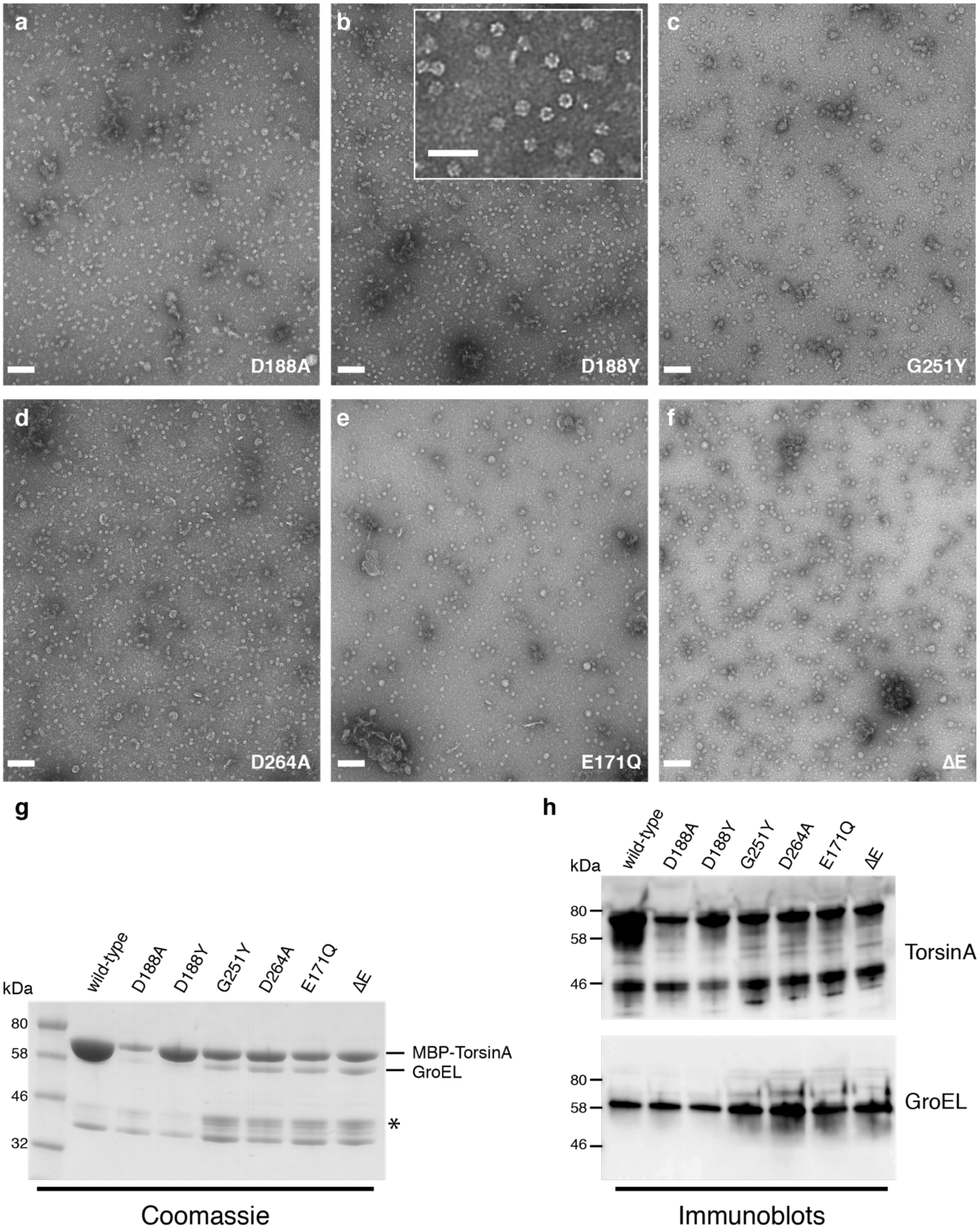
a
D188A
b
D188Y
c
G251Y
d
D264A
e
E171Q
f
ΔE
g
kDa
wild-type
D188A
D188Y
G251Y
D264A
E171Q
ΔE
80
58
46
32
MBP-TorsinA
GroEL
*
Coomassie
h
kDa
wild-type
D188A
D188Y
G251Y
D264A
E171Q
ΔE
80
58
46
TorsinA
80
58
46
GroEL
Immunoblots

Supplementary Figure 5. Mutational analysis of TorsinA filaments

Negative stain electron micrographs of the TorsinA mutants (**a**) D188A, (**b**) D188Y, (**c**) G251Y, (**d**) D264A, (**e**) E171Q, and (**f**) ΔE. No filaments were observed regardless of the buffer conditions. The protein eluates were contaminated with bacterial GroEL which we observe on micrographs. The inset shows a higher magnification view of (**b**), where the contaminant GroEL is noticeable. Scale bar is 100 nm in all micrographs, and 50 nm in the inset. To validate the presence of GroEL contamination independently, and to determine its relative amount compared to the TorsinA variants, equal volumes of the purified samples were also analyzed by SDS-PAGE (**g**), and immunoblotting (**h**). Compared to wildtype, more GroEL contamination was observed for most of the TorsinA mutants. Asterisk denotes a truncated form of TorsinA which we consistently observe in our amylose elutes.

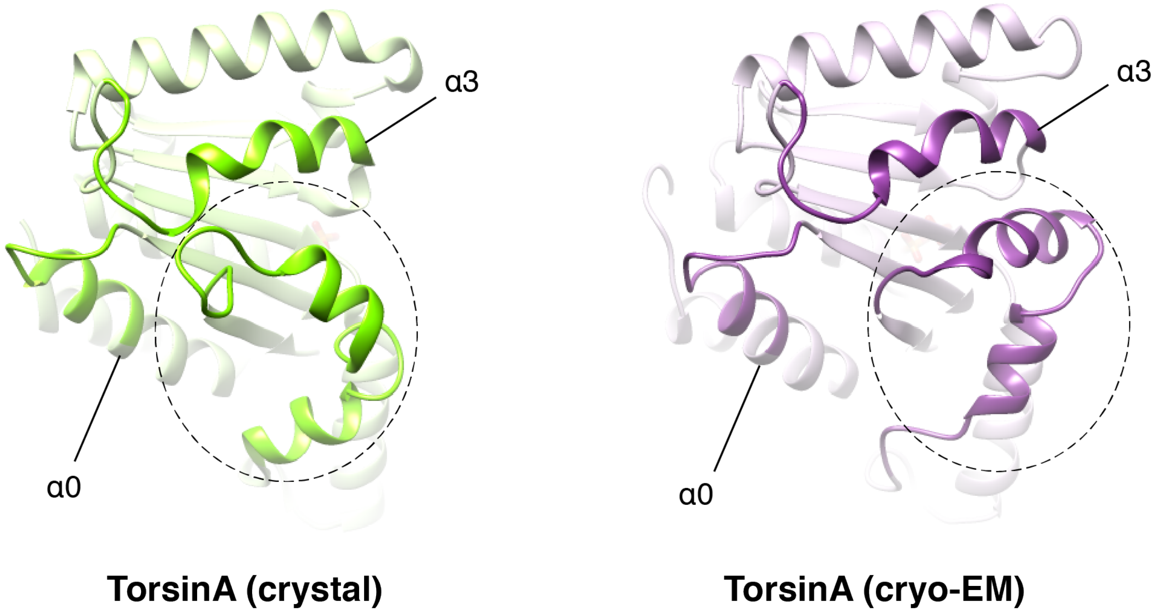

Supplementary Figure 6. The ‘backside’ of TorsinA in the LULL1-bound and filamentous states

The non-catalytic, nucleotide-free face of TorsinA is displayed in the LULL1-bound, crystallized form (left) and the filamentous form (right). Structural elements residing at the subunit-subunit interface of the TorsinA polymer are shown in dark purple. The same elements are shown in dark green in the crystal structure. The rearranged region of TorsinA (aa 232-262) is marked with a circle.

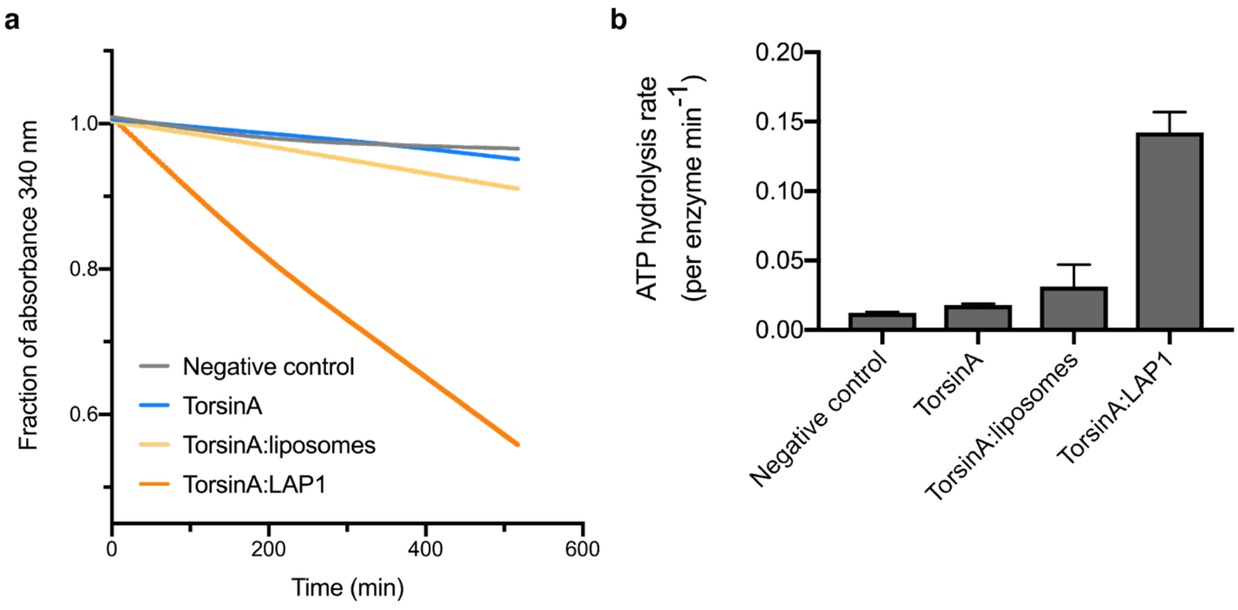

Supplementary Figure 7. ATPase assays

(**a**) ATPase activity of TorsinA filaments, TorsinA:LAP1 and TorsinA:liposome mixes were measured in an NADH-coupled ATPase assay, as previously described [1,2]. Shown are the oxidation rate plots, from where the ATP hydrolysis rates were calculated using linear regression. The negative control contains all of the components of the assay except TorsinA. (**b**) Bar graph representation of the ATPase assay results shown in (a). Error bars indicate SEM.

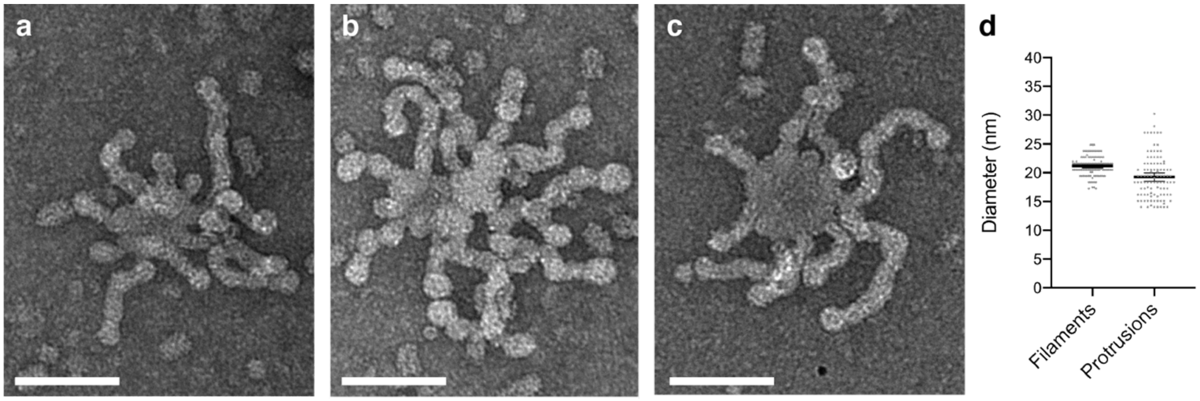

Supplementary Figure 8. MBP-TorsinA tubulates membranes *in vitro*

(**a**-**c**) Negative stain images of liposomes which are tubulated after incubation with MBP-TorsinA for 6 hours at RT. Scale bar, 100 nm. (**d**) Scatter plot of the diameters measured from negatively stained micrographs using ~100 MBP-TorsinA filaments and ~100 lipid protrusions. Mean values are shown together with 95% confidence intervals.

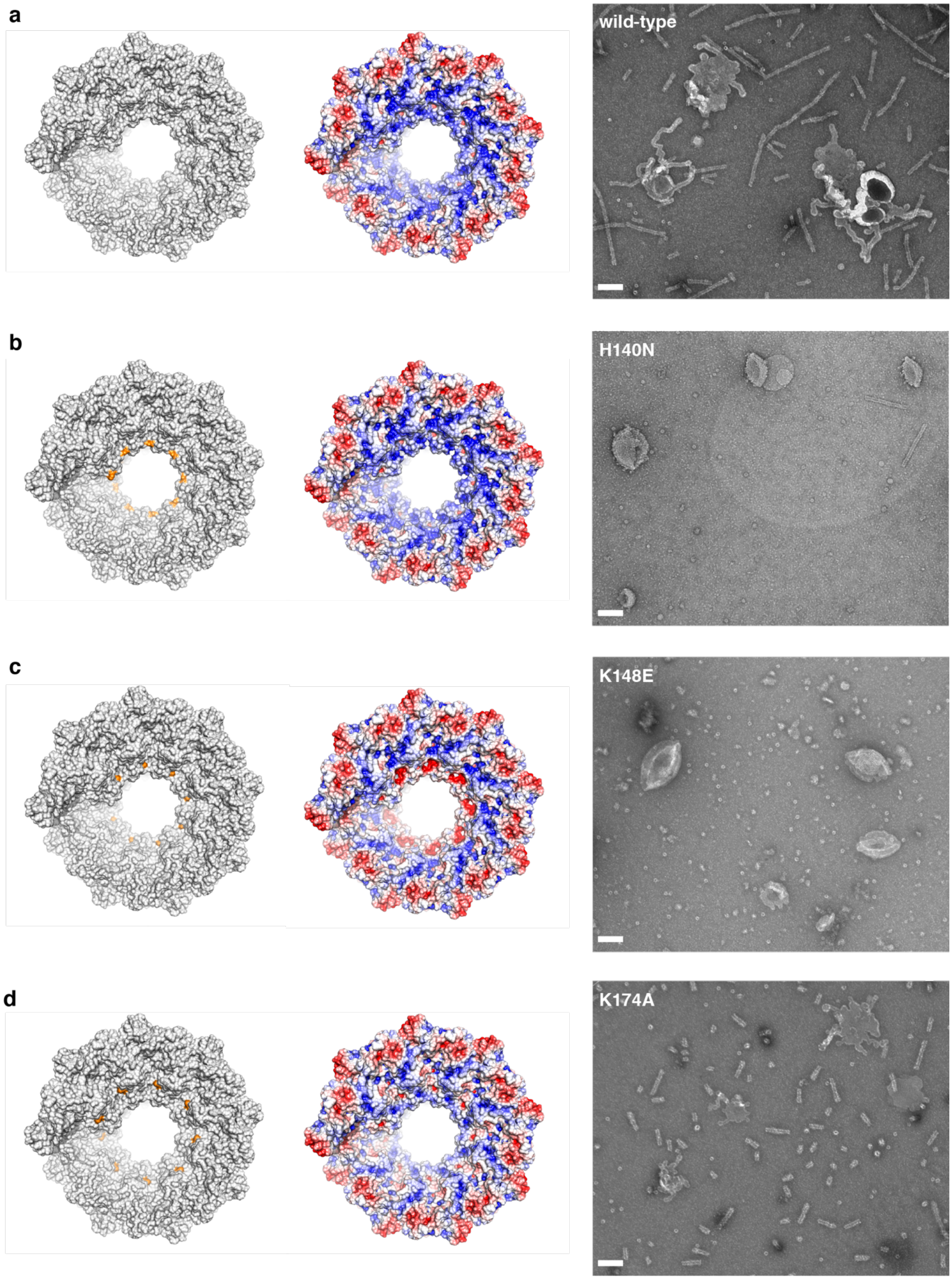

a
wild-type
b
H140N
c
K148E
d
K174A

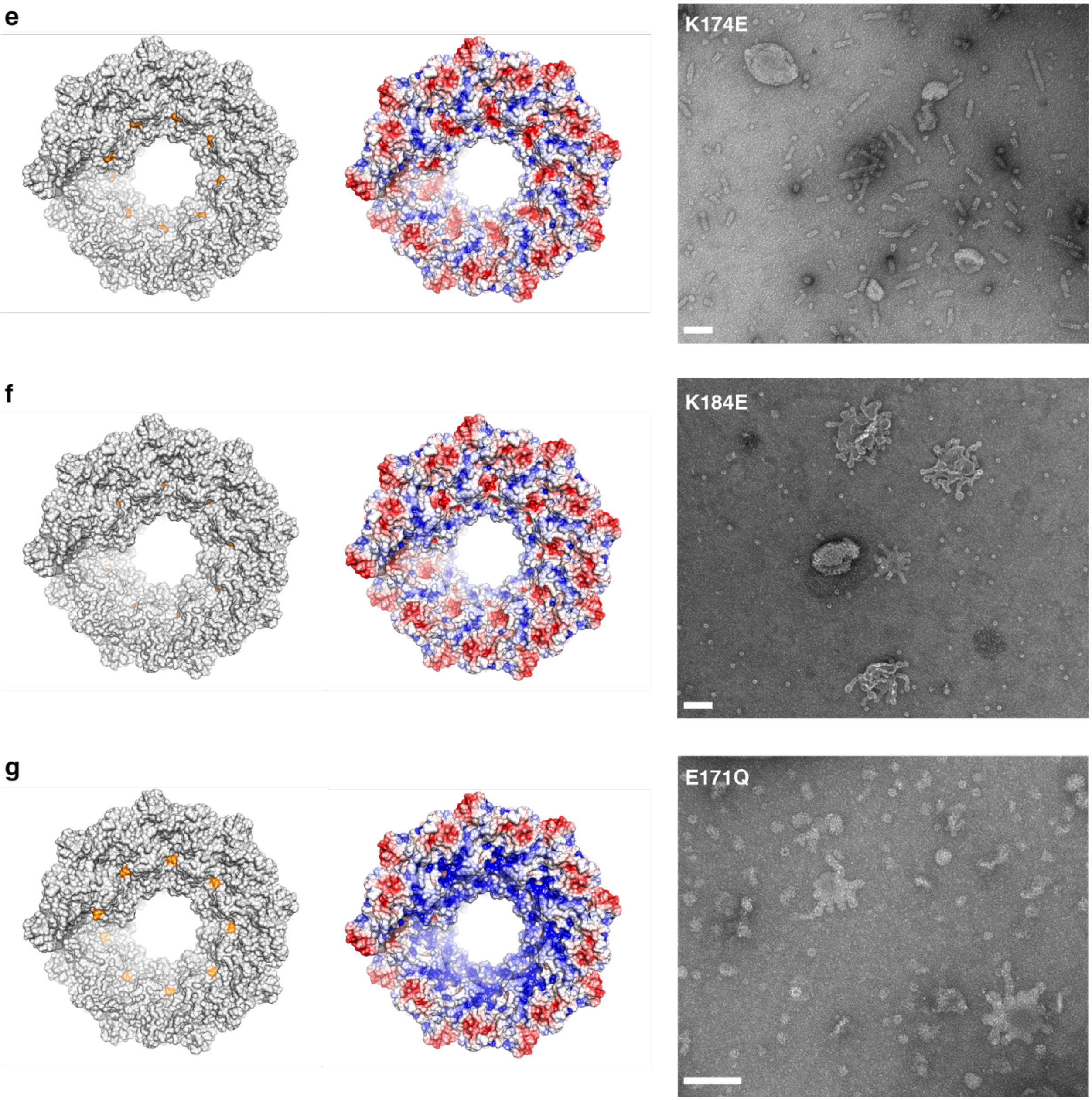

Supplementary Figure 9. Mutational analysis of liposome tubulation

Negative stain images of liposomes after incubation with the TorsinA variants (**a**) wild-type, (**b**) H140N, (**c**) K148E, (**d**) K174A, (**e**) K174E, (**f**) K184E, and (**g**) E171Q. Except for the ATP-trap mutation E171Q, all other residue substitutions targeted the inner channel surface of the TorsinA filaments at highly conserved, mostly positively charged residues. Mutated residues are marked in orange on an end-view of the TorsinA filaments (left panels), and the electrostatic

surface of the each TorsinA mutant is shown in same view (middle panels), next to the micrographs (right panels). H140N, K148E, and K174E mutations prevent tubulation of liposomes. The mutants K148E, K184E and E171Q do not form filaments in solution. Scale bar, 100 nm.

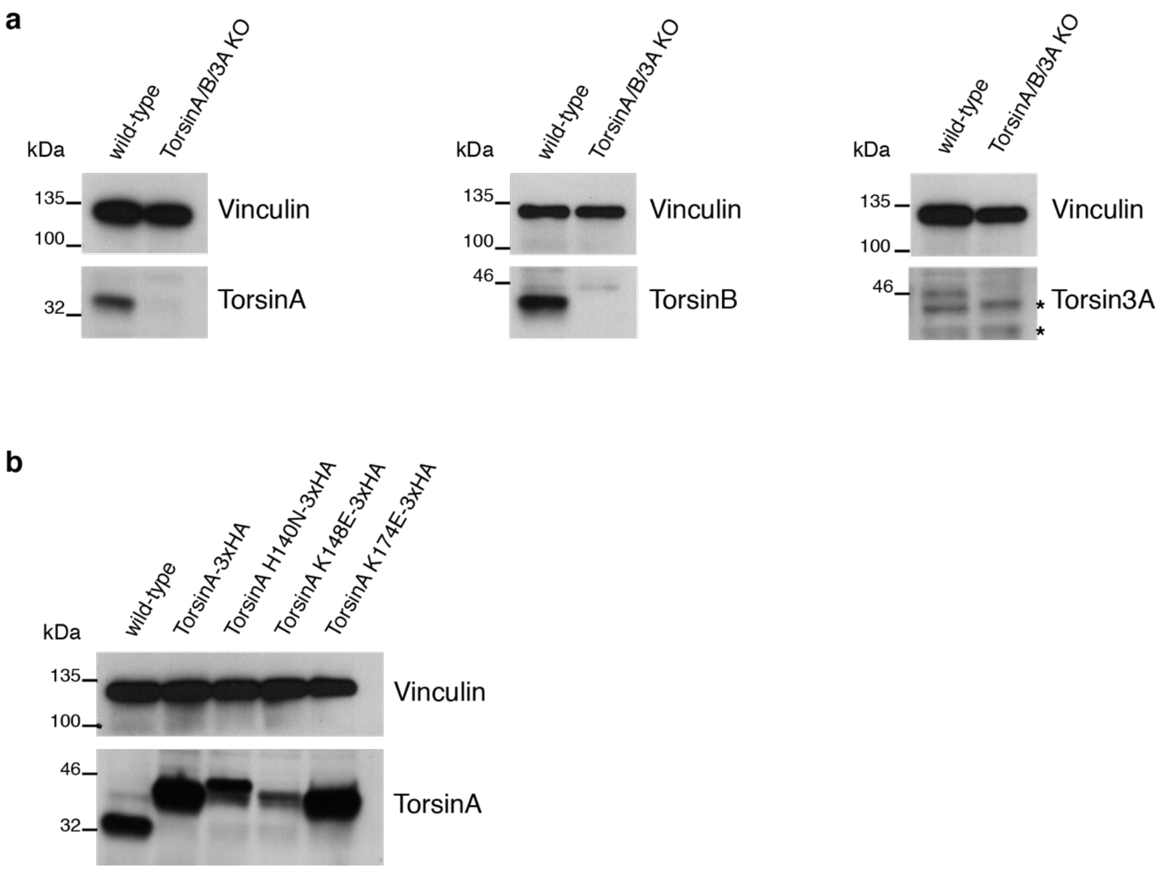

Supplementary Figure 10. Generation of HeLa cell lines

(**a**) Immunoblots of the wild-type and the TorsinA/B/3A knockout cell lysates using an anti-TorsinA (left), an anti-TorsinB (middle), or an anti-Torsin3A (right) antibody. Asterisks denote nonspecific background bands. Loading control is vinculin. (**b**) Immunoblots of the rescue cell lines expressing TorsinA-3xHA variants in the TorsinA/B/3A knockout background, in comparison to wild-type cells. Anti-TorsinA antibody is used, and the loading control is vinculin.

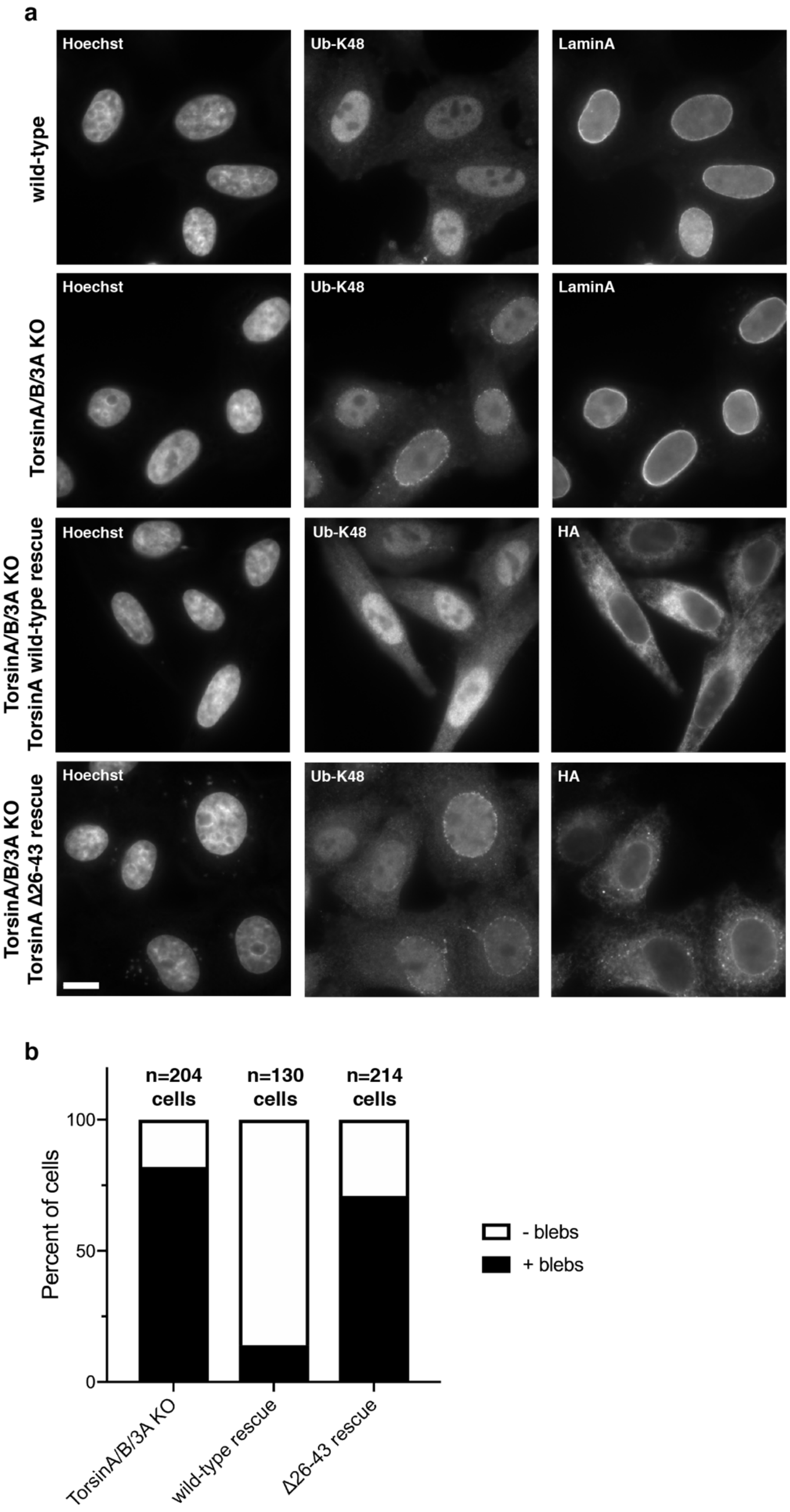

a
Hoechst
Ub-K48
LaminA
HA
wild-type
TorsinA/B/3A KO
TorsinA/B/3A KO TorsinA wild-type rescue
TorsinA/B/3A KO TorsinA Δ26-43 rescue
b
n=204 cells
n=130 cells
n=214 cells
Percent of cells
100
50
0
- blebs
+ blebs
TorsinA/B/3A KO
wild-type rescue
Δ26-43 rescue

Supplementary Figure 11. The N-terminal hydrophobic portion of TorsinA is necessary to rescue nuclear blebbing in TorsinA/B/3A KO cells.

(**a**) Immunofluorescence images of the wild-type HeLa cells, TorsinA/B/3A KO cells, and the TorsinA/B/3A KO cells rescued by transient expression of either the wild-type TorsinA-HA or the TorsinA Δ26-43-HA constructs. DNA is visualized using Hoechst, the nuclear rim is marked with an anti-Lamin A antibody. Wild-type TorsinA-HA and the TorsinA Δ26-43-HA mutant are stained with an anti-HA antibody. Nuclear blebs are marked as K48-Ubiquitin foci using an anti-K48 Ubiquitin antibody [3]. Scale bar, 10 μm. (**b**) Percentage of TorsinA/B/3A KO cells showing nuclear blebs with or without the expression of the wild-type TorsinA-HA and the TorsinA Δ26-43-HA rescue constructs.

Supplementary References

1. Sosa, B. A. *et al.* How lamina-associated polypeptide 1 (LAP1) activates Torsin. *Elife* **3,** e03239 (2014).
2. Nørby, J. G. Coupled assay of Na+,K+-ATPase activity. *Meth. Enzymol.* **156,** 116–119 (1988).
3. Laudermilch, E. *et al.* Dissecting Torsin/cofactor function at the nuclear envelope: a genetic study. *Mol. Biol. Cell* mbc.E16–07–0511 (2016). doi:10.1091/mbc.E16-07-0511
