# Supplementary material for: The AAA + ATPase TorsinA polymerizes into hollow helical tubes with 8.5 subunits per turn
Source: Nat Commun. 2019 Jul 22;10:3262. doi: 10.1038/s41467-019-11194-w (PMC6646356; doi:10.1038/s41467-019-11194-w)
Supplement: Supplementary file 3 — Reporting Summary [file 41467_2019_11194_MOESM3_ESM.pdf]

nature research

Corresponding author(s): Thomas U. Schwartz

Last updated by author(s): Jun 6, 2019

# Reporting Summary

Nature Research wishes to improve the reproducibility of the work that we publish. This form provides structure for consistency and transparency in reporting. For further information on Nature Research policies, see Authors & Referees and the Editorial Policy Checklist.

## Statistics

For all statistical analyses, confirm that the following items are present in the figure legend, table legend, main text, or Methods section.

| n/a | Confirmed | |
|---|---|---|
| ☐ | ☒ | The exact sample size (*n*) for each experimental group/condition, given as a discrete number and unit of measurement |
| ☐ | ☒ | A statement on whether measurements were taken from distinct samples or whether the same sample was measured repeatedly |
| ☒ | ☐ | The statistical test(s) used AND whether they are one- or two-sided<br>*Only common tests should be described solely by name; describe more complex techniques in the Methods section.* |
| ☒ | ☐ | A description of all covariates tested |
| ☒ | ☐ | A description of any assumptions or corrections, such as tests of normality and adjustment for multiple comparisons |
| ☐ | ☒ | A full description of the statistical parameters including central tendency (e.g. means) or other basic estimates (e.g. regression coefficient) AND variation (e.g. standard deviation) or associated estimates of uncertainty (e.g. confidence intervals) |
| ☒ | ☐ | For null hypothesis testing, the test statistic (e.g. *F*, *t*, *r*) with confidence intervals, effect sizes, degrees of freedom and *P* value noted<br>*Give P values as exact values whenever suitable.* |
| ☒ | ☐ | For Bayesian analysis, information on the choice of priors and Markov chain Monte Carlo settings |
| ☒ | ☐ | For hierarchical and complex designs, identification of the appropriate level for tests and full reporting of outcomes |
| ☒ | ☐ | Estimates of effect sizes (e.g. Cohen's *d*, Pearson's *r*), indicating how they were calculated |

*Our web collection on statistics for biologists contains articles on many of the points above.*

## Software and code

Policy information about availability of computer code

| | |
|---|---|
| Data collection | Electron cryomicroscopy: SerialEM 3.7 beta |
| Data analysis | Helical reconstruction: AlignFrames (IMOD), CTFFIND3, e2helixboxer (EMAN2), SPIDER; Model building and refinement: UCSF Chimera, Coot, RosettaCM, PHENIX, MolProbity; Structural analysis: PDB2PQR, APBS, ConSurf, PDBePISA, PyMOL; ATPase activity assay and the diameter analysis of the filaments/lipid protrusions: Prism8 (GraphPad). |

For manuscripts utilizing custom algorithms or software that are central to the research but not yet described in published literature, software must be made available to editors/reviewers. We strongly encourage code deposition in a community repository (e.g. GitHub). See the Nature Research guidelines for submitting code & software for further information.

## Data

Policy information about availability of data

All manuscripts must include a data availability statement. This statement should provide the following information, where applicable:

- Accession codes, unique identifiers, or web links for publicly available datasets
- A list of figures that have associated raw data
- A description of any restrictions on data availability

All data and the biologically unique materials generated in this study are available from the corresponding author upon request. A reporting summary for this article is available as a Supplementary Information file. The atomic coordinates for the TorsinA structure have been deposited in the Protein Data Bank (PDB) under the accession code 6OIF, and the corresponding EM density map has been deposited to the Electron Microscopy Data Bank (EMDB) under the accession code EMD-20076. The source data underlying Figs. 3,4, and Supplementary Figs. 5,7,8,10 are provided as a Source Data file.

# Field-specific reporting

Please select the one below that is the best fit for your research. If you are not sure, read the appropriate sections before making your selection.

☒ Life sciences ☐ Behavioural & social sciences ☐ Ecological, evolutionary & environmental sciences

For a reference copy of the document with all sections, see nature.com/documents/nr-reporting-summary-flat.pdf

# Life sciences study design

All studies must disclose on these points even when the disclosure is negative.

| | |
|---|---|
| Sample size | Cryo-EM micrographs used for helical reconstruction were selected based on the CTF estimation and defocus < 3 µm calculated by the software CTFFIND3. Lipid protrusions and filaments in negative stain electron micrographs were manually counted and measured for diameter analysis. Likewise, in fluorescence microscopy images, cells containing K48-Ubiquitin foci denoting the nuclear blebs were manually counted. |
| Data exclusions | No data were excluded from the analyses. |
| Replication | All attempts at replication were successful. |
| Randomization | Experimental conditions are defined in the paper. No randomization was necessary. |
| Blinding | Investigators were blinded to the sample type during image acquisition of the EM cross-sections obtained from the cell lines expressing different TorsinA-3xHA variants. Blinding was not possible otherwise, since the investigator performing the analysis also contributed to isolation of the specimen being analyzed. |

# Reporting for specific materials, systems and methods

We require information from authors about some types of materials, experimental systems and methods used in many studies. Here, indicate whether each material, system or method listed is relevant to your study. If you are not sure if a list item applies to your research, read the appropriate section before selecting a response.

## Materials & experimental systems

| n/a | Involved in the study |
|---|---|
| ☐ | ☒ Antibodies |
| ☐ | ☒ Eukaryotic cell lines |
| ☒ | ☐ Palaeontology |
| ☒ | ☐ Animals and other organisms |
| ☒ | ☐ Human research participants |
| ☒ | ☐ Clinical data |

## Methods

| n/a | Involved in the study |
|---|---|
| ☒ | ☐ ChIP-seq |
| ☒ | ☐ Flow cytometry |
| ☒ | ☐ MRI-based neuroimaging |

## Antibodies

| | |
|---|---|
| Antibodies used | Mouse monoclonal D-M2A8 against TorA (a gift from Cristopher Bragg, Massachusetts General Hospital/Harvard); rabbit anti-TorB (a gift from Rose Goodchild, VIB-KU Leuven Center for Brain & Disease Research, Leuven, Belgium); rabbit polyclonal anti-Tor3A (ARP33117_P050, Aviva Systems Biology); mouse monoclonal anti-vinculin (ab130007, Abcam); rabbit anti-GroEL (G6532, Sigma-Aldrich), goat anti-mouse IgG-HRP (sc-2055, Santa Cruz Biotechnology); goat anti-rabbit IgG-HRP (7074P2, Cell Signaling Technology); rabbit anti-K48 Ubiquitin (05-1307, Millipore); mouse anti-Lamin A (ab8980, Abcam); mouse anti-HA (H9658, Sigma-Aldrich); anti-mouse Cy3 (715-165-150, Jackson ImmunoResearch) and anti-rabbit Cy2 (711-225-152, Jackson ImmunoResearch). |
| Validation | All primary antibodies were validated by immunoblot detection of a specific band at the expected molecular weight in lysates obtained from wild-type HeLa cells. Validation for the primary antibodies used in immunofluorescence experiments are as follows:<br>rabbit anti-K48 Ubiquitin (05-1307, Millipore): http://www.emdmillipore.com/US/en/product/Anti-Ubiquitin-Antibody-Lys48-Specific-clone-Apu2-rabbit-monoclonal,MM_NF-05-1307#overview<br>mouse anti-Lamin A (ab8980, Abcam): https://www.abcam.com/lamin-a-antibody-133a2-ab8980.html<br>mouse anti-HA (H9658, Sigma-Aldrich): https://www.sigmaaldrich.com/catalog/product/sigma/h9658?lang=en&region=US<br>rabbit anti-GroEL (G6532, Sigma-Aldrich): https://www.sigmaaldrich.com/catalog/product/sigma/g6532?lang=en&region=US |

## Eukaryotic cell lines

Policy information about cell lines

| | |
|---|---|
| Cell line source(s) | HeLa and 293-GP cell lines were in the Cheeseman lab's cell bank (Massachusetts Institute of Technology) and were obtained by former lab members. Other cell lines generated in this study were derived from these sources. |
| Authentication | Verification has not been performed on the HeLa source cell line, and cell type was not relevant for our conclusions. |
| Mycoplasma contamination | Cell cultures were checked every couple of months for mycoplasma contamination using Mycoalert (Lonza). |
| Commonly misidentified lines (See ICLAC register) | No commonly misidentified cell lines were used in this study. |
